# Research on the impact of clan network on farmers’ entrepreneurial income—The case of China

**DOI:** 10.3389/fpsyg.2022.951421

**Published:** 2022-10-04

**Authors:** Xiaoli Jiang, Qianwen Wu, Lina Wang, Beirui Jiang, Xiao Ma

**Affiliations:** ^1^College of Marxism, Minjiang University, Fuzhou, China; ^2^School of Economics and Management, Fujian Agriculture and Forestry University, Fuzhou, Fujian, China; ^3^Newhuadu Business School, Minjiang University, Fuzhou, Fujian, China

**Keywords:** clan network, farmer entrepreneurship, social capital, entrepreneurial income, informal institutions

## Abstract

Farmers’ entrepreneurial income is related to poverty alleviation and common prosperity. The clan network is an important social capital for farmers. However, research on effects of this relationship is still scant. We classifies farmers’ social capital as endowed social capital such as clan networks and leapfrogging social capital that needs to be operated. Using the data of CFPS 2010–2018 in China, this study investigates the influence of clan networks and farmers’ entrepreneurial income. Based on the social capital theory, we adopt a semilogarithmic model, and propensity score matching method for robustness checks. The results show that the clan network, as an endowed social capital of farmer, has a significant and positive effect on entrepreneurial income for both men and women. And the clan network has the greatest impact on middle-income farmers. Our results have important implications for policymakers in other developing economies who seek to increase farmers’ income.

## Introduction

It is obvious that entrepreneurial activities have made outstanding contributions to China’s economic transformation and upgrading (Yin et al., 2019). More importantly, establishing and supporting farmer entrepreneurship have become a necessity to achieve sustainability, alleviate poverty, and revitalize the rural economy (Savrul, 2017; Naminse et al., 2019). Farmers’ entrepreneurial income is related to the high quality of entrepreneurship and the improvement of entrepreneurial ability. In general, ideal farmers’ entrepreneurial income has a positive effect on alleviating farmers’ poverty, improving farmers’ well-being, and promoting comprehensive rural revitalization. Therefore, understanding the influencing factors of farmers’ entrepreneurial income has attracted increasing attention. When the background is Chinese peasant entrepreneurship, one of the most important social organizations of Chinese family groups cannot be ignored (Greif and Tabellini, 2017). So, we pay particular attention to the set of informal institutions that could affect members’ entrepreneurial behavior, the more specific point is the clans, which are one of the most important vehicles of informal institutions in rural China (Jiang et al., 2022).

At present, there are few studies on the influence of clan networks on farmers’ entrepreneurial income from the perspective of social capital. Generally speaking, farmer entrepreneurs in emerging economies usually choose labor-intensive industries that require a small capital scale and a low technical threshold (Deller et al., 2019; Miao et al., 2021). The subjectivist theory of entrepreneurship suggests entrepreneurial behavior as a function of differences in knowledge, resources, opportunity costs, and expectations between founders (Kor et al., 2007). The clan network is an important social capital for farmers due to its characteristics of reciprocity, mutual forbearance and trust.

As the social capital of farmers, what impact does clan network have on farmers’ entrepreneurial income? In the short run, social capital can alleviate the financial constraints of farmers’ entrepreneurship, and bring more sources of information, which helps to alleviate information asymmetry. However, does social capital based on the pattern of difference sequence (Fei, 2009) influence entrepreneurship to increase to another level and widen the entrepreneurial income gap? Is social capital for the poor? What is the relationship between social capital and the process of marketization?

Based on the above-unanswered questions, this paper regards the clan network as the farmers’ endowment-type social capital, an informal system, and argues that the inconclusive disagreement on the above questions lies in the fact that no classification of individual social capital has been made.

To study the questions mentioned above, this paper mainly carried out the following work: First, we discusses the impact of clan networks as endowed social capital and leapfrog social capital on farmers’ entrepreneurial income and provides a possible explanation of whether clan social capital is entirely poor people’s capital and what kind of social capital is the social capital of the poor. Second, it analyzes the relationship between the clan network as an informal system and the influence of the formal system on farmers’ entrepreneurial income, addressing the question of the relationship between the informal system and the marketization process.

This paper makes the following contributions to the literature. First, this study enriches the theoretical research on social capital with Chinese characteristics. We devide the social capital possessed by farmers into endowment-type social capital (clan network) and leapfrog-type social capital (social relations), and answer the question does social capital belong to the poor. The paper argues that in the process of marketization, the two types of social capital play different roles and have different effects on increasing entrepreneurial income and jointly support the increase of farmers’ entrepreneurial income.

Second, the paper expands the research on the institutional field of New Institutional Economics by analyzing the influence of informal institutions on farmers’ entrepreneurial behavior. It also clarifies the relationship between the formal market system and the informal clan system in the context of China, and analyzes the mutual construction mechanism and evolution law between the rural society and the market economy. As an informal system, the clan network has an “inverted U-shaped” relationship between its role and the development level of marketization. When the market system is gradually strengthened, the influence of the clan network as an informal system in farmers’ entrepreneurial activities gradually fades. In the period of economic transformation, the study of how the informal system of clan networks affects farmers’ entrepreneurial income will help to clarify the traditional and modern interactive games and relationship embedding in the process of institutional transformation to understand institutional transformation more comprehensively.

To achieve these goals, the rest of the paper is divided as follows. Section “Literature Review” and “Data and Methodology,” we demonstrate our method of data collection, variable selection and descriptive analysis. Section “Empirical Results and Discussion,” “Conclusions” and “Policy Implications and theoretical implication“. We present the followings.

## Literature review

### Clan network

A promising and fruitful line of research seeks to explain the definition and influencing mechanism of Chinese clan groups. The traditional Chinese economic structure is characterized by the clan as the material carrier of the extended family and the realization of various relationships, such as clan house branches, collateral relatives, friends, and classmates, which constitute the basic relationships within the system. As the scope of social interaction expands, various relationships ensue, constituting the basic interpersonal relationships (Luo and Gao, 2011). Clans have network that are formed by blood relationships, which may share job information with unemployed members or help them find jobs directly (Foltz et al., 2020). The clan networks may also help members overcome financial constraints and establish businesses (Kinnan and Townsend, 2012; Zhang, 2020). Since clans exist in many places, especially Asian countries such as Singapore (Freedman, 1958), Korea (Yang, 2019), and Philippines (Cruz et al., 2020), the study has general implications beyond China.

The connotation of the transactional relationship in the clan network includes trust and the evolution of cooperative relationships. The clan network gives special attention to the concepts of reciprocity, mutual forbearance, and trust (He et al., 2017). The clan networks are highly important institutions since they facilitate cooperation (Greif and Tabellini, 2017). Therefore, a clan network is a form of social capital, and its core content can be defined as characteristics of social organization, such as trust, norms and networks (Putnam, 1994). In the clan network, members belong to a society of acquaintances and have a high degree of mutual trust. Process-based trust is embedded in specific clan networks, and reciprocity in return is a key mechanism for maintaining trust between groups. Mutual trust underpins a reciprocal process that provides the potential social capital to be mobilized. In this sense, clan networks are a special kind of endowed social capital.

As a special kind of endowed social capital, clan networks play a role in coordinating and complementing villagers’ autonomous organizations to a certain extent. For example, they can supplement and coordinate the work of village committees in mediating civil disputes, maintaining public safety, and developing culture and education. In addition, clan networks have played an active role in helping low-productivity farmers or poor people find outside work to increase off-farm income, thereby helping to narrow the income gap in rural areas. With the acceleration of the reform and opening-up process, the role of clan networks in narrowing the income gap has become increasingly stronger (Guo et al., 2014).

One of the motivations that drives people to engage in economic activities is not only to obtain the satisfaction of material interests but also to fulfill emotional needs such as family affection, friendship, dignity, and recognition in economic exchanges. In other words, both material benefits and spiritual needs can be obtained in economic transactions. The driving force is relatively deep and fundamental. Similarly, the social capital in the hands of clan members drives members to help each other and overcome obstacles to entrepreneurship.

Social capital in clan networks can be based on a continuum between affective and instrumental components, and relationships can be classified as affective-type, hybrid-type, and instrumental-type. Alternatively, according to the degree of responsibility, relationships can also be classified as coercive, reciprocal, and utilitarian, which can reduce knowledge hiding behaviors (He et al., 2020). Most of the relationships among clan members are emotional, possess no deliberate management and are a kind of endowed social capital. However, outside the clan network circle, other relationships need to be managed deliberately, mixed with mutual benefit and reciprocity, and belong to leapfrog social capital. The process-based trust mosaic in a specific clan network, on the one hand, strengthens communication and exchange among network members through frequent social activities and other forms, prompting entrepreneurs to obtain rich entrepreneurial information and knowledge, thus alleviating entrepreneurial information asymmetry and helping entrepreneurs gain insight into business activities and market dynamics. On the other hand, network members can help solve dilemmas encountered in the entrepreneurial process while facilitating entrepreneurs to expand sales channels, thus affecting entrepreneurial income. At present, the research on the influence of clan networks on farmers’ entrepreneurship is not abundant enough, while that on the farmers’ entrepreneurship performance is even less.

### The link between clan networks and farmers’ entrepreneurial income

Research on farmer entrepreneurship in emerging countries shows that, entrepreneurs create greater socioeconomic benefits for local communities while realizing their own development through formalization (Yessoufou et al., 2017). The study of factors influencing farm household entrepreneurial income include farmer trait theory, resource-based theory, and institutional environment theory. Farmer trait theory considers some farmers’ personalities, such as autonomy, honesty, aggressiveness, and willpower, as core traits that influence the success of farmers’ entrepreneurship (Zhao and Zhou, 2006; Marineau and Nordstrom, 2020). Farmer entrepreneurs’ extroversion and openness to experience traits are conducive to exploratory learning, and the higher their scores on extroversion, emotional stability, due diligence, and openness to experience traits, the better their utilization-based learning and the better their entrepreneurial income (Luo and Chen, 2014). The overall entrepreneurial ability affects farmers’ entrepreneurial income (Cai et al., 2014; Xie and Huang, 2016).

Agency theory and resource-based theory (Chrisman et al., 2005) focus on factors such as physical capital, political capital, human capital, and social capital under the framework of sustainable livelihoods. For example, Saxton et al. (2016) divided entrepreneurial support behaviors into three categories: providing products/services, human resources, and financial resources, and expounded the roles of various support behaviors. Xu (2008) found that land fragmentation induced by the household contract responsibility system was a cause of the widening income gap among farm households. In addition, a large body of literature finds that education, as the most dominant human capital variable, enhances entrepreneurship significantly. Plausible mechanisms that drive our results are resource acquisition, opportunity identification, and decrease in labor cost (Qin and Kong, 2021). However, the impact of human capital on farmers’ entrepreneurial income has not yet reached a consensus conclusion from the empirical evidence due to the controversy over the measurement indicators of both. The points of interest for the influencing factors of entrepreneurial income are that education level, training status, the experience of working outside the home, and entrepreneurial experience are significantly and positively related to the choice of entrepreneurial industry and entrepreneurial income of both father and son farmer entrepreneurs (Luo and Huang, 2014). Human capital reflected by education level and on-the-job training is the main reason for widening the income gap of farmers (Gao and Yao, 2006). Dutta and Russell (2018) argued that a positive effect of human capital on actual startup activity can be found, with an explanatory path of entrepreneurial human capital influencing the level of entrepreneurial social capital and thus the performance of the venture (Mosey and Wright, 2007; Li et al., 2021; Xie et al., 2021) and another explanatory path of better performance of new ventures when the human capital of the entrepreneurial team matches the corporate strategy (Shrader and Siegel, 2007).

Zhao and Lu (2010) examined the impact of social networks (especially Guanxi) on income and concluded that the role of social networks in raising income and the contribution of social networks to income disparity are significantly higher in the eastern region, where the degree of marketization and the level of economic development are higher than in the central and western regions. At the same time, business network nesting (Ceci et al., 2019) is an important factor affecting farmers’ entrepreneurial performance. Based on the framework of sustainable livelihood, the six-dimensional entrepreneurial capital system of natural capital, psychological capital, human capital, social capital, material capital, and financial capital has a significant positive impact on entrepreneurial income, including entrepreneurial self-efficacy, optimism, the number of family laborers, the number of formal financial institutions in the region, the credit relationship with credit institutions, the purchase of transportation expenses, family business income (Su et al., 2016).

Overall, existing research focuses on the factors that influence farmers’ entrepreneurial income in terms of prior knowledge and experience, human capital endowment, social capital endowment, and entrepreneurial environment (Zhu and Xie, 2012; Zhang and Tang, 2013; Zhou, 2013) and focuses on the relationship between entrepreneurial opportunity discovery, entrepreneurial motivation, entrepreneurial ability, social capital, intergenerational inheritance, policy resource acquisition and farmers’ entrepreneurial income. However, some studies have also shown that social networks do not absolutely play a positive role in the development of farmers’ entrepreneurship. Dong et al. (2018) found that the relationship between friends and relatives in social networks can increase farmers’ mental and financial burdens, thus hindering the income enhancement of farmers’ entrepreneurial activities.

In recent years, scholars have investigated how institutions affect entrepreneurship and innovation. How the institutional environment and its changes affect the governance structure, resource allocation, strategic decisions, and performance levels of firms (North and Hang, 2008; Peng et al., 2008). For small and medium-sized family firms that are vulnerable to institutional change, the particular institutional context of transition is one of the important factors determining the level of firm strategy and performance, and they are also more sensitive to changes in the external institutional environment (Yang and Li, 2008). He et al. (2016) show that in regions where the rate of institutional change is slow, an acceleration in the rate of institutional change leads family business decision-makers to reduce unproductive activities and increase investment in productive activities.

The existing institutional context research is carried out on the interaction between institutional context and entrepreneurial income, but there is a lack of in-depth research on how entrepreneurs or entrepreneurial organizations adapt to a certain institutional context. In addition to formal institutions such as laws and policies, entrepreneurship is also influenced by informal institutions such as cultural traditions, popular opinion, and religion (Tian and Wang, 2016). Formal and informal institutions may sometimes have a serial impact on the strategic actions of firms (Yong et al., 2021).

Therefore, the actions taken by entrepreneurs and the entrepreneurial strategies adopted by enterprises have been comprehensively affected by various formal institutions. For example, formal institutions factors such as government support and loan ease have the most significant effect on farmers’ entrepreneurial income, followed by the second influencing factors of the human capital factor and psychological quality factor, while the formal institutions has a more general effect on farmers’ entrepreneurial income (Zhang and Tang, 2013).

The angle of influencing factors on farmers’ entrepreneurial income, which is closer to the research focus of this paper, mainly focuses on social capital, which primarily refers to the resources embedded in the social network acquired and used by actors in their actions (Cai et al., 2021; Xie et al., 2021). Studies have also found that trust is significantly and positively related to the business performance of farmer entrepreneurs, and entrepreneurial learning plays a significant mediating role between trust and entrepreneurial performance (Zhao et al., 2020). The above research shows that social networks affect farmers’ entrepreneurial income gap (Wang W. et al., 2019), but more rigorous empirical evidence is still needed. The social network proxy variables used in the current study are the number of friends and relatives working in the government sector or the number of peers who can help when the individual is looking for a job. Although the proxy variables are somewhat representative of the social networks, there is still a subtle problem: these social networks can have weak relationships and thus no impact, leading to biased estimates.

A few relevant studies focus on the environmental support of clan networks for farmers’ entrepreneurship and the level of innovative entrepreneurship. Lin et al. (2019) argue that clan networks provide important economic and social capital for industrial development, supporting local enterprises in important aspects such as land acquisition and financing. Dong et al. (2019) concluded that overall the influence of clan size on the innovation level of rural entrepreneurial firms was not significant, while clan intensity had a significant positive effect on the innovation level of firms. The positive influence of clan strength is also more prominent in areas with high levels of economic development or strong clan culture. However, most of the current literature does not consider the impact of clan networks as the important social capital of farmers on the income gap of farmers’ entrepreneurship, the impact of different types of capital, and the differences of this impact in different stages of marketization and regions.

## Data and methodology

### Data sources

To explore the factors affecting farmers’ entrepreneurial income and to specify the impact of endowed social capital, leapfrog social capital, human capital, political capital, and other types of capital on farmers’ entrepreneurial income, the main data were obtained from the China Family Panel Studies (CFPS) 2010, 2012, 2014, 2016, and 2018 survey data from the China Social Science Survey Center of Peking University. The CFPS questionnaire consists of a community questionnaire, family questionnaire, adult questionnaire and children’s questionnaire, covering personal information such as work status, education level, cognitive ability, personality characteristics, and entrepreneurship.

The formal system uses China’s marketability index as a proxy variable. China Marketability Index data were obtained from the China Sub-Provincial Enterprise Business Environment Index 2017 Report (Wang et al., 2017) and the China Sub-Provincial Marketability Index Report (2017) (Wang X. et al., 2019), which contains the business environment index for each province and city in 2016, 2010 and 2012 (the 2018 data were not publicly published at the time of writing the main body of the paper, and this paper populates the 2018 data using trends). The market index includes the following composite scores: policy openness, equity and justice, administrative intervention and government integrity, the legal environment for business operations, corporate taxation, financial services and financing costs, human resource availability, infrastructure conditions, and intermediary services. The report uses quantitative indicators for cross-sectional comparisons to reflect the differences in the business environment of each company and to identify the main factors affecting the business environment and the characteristics of each company’s business environment.

### Variable selection and descriptive analysis

The sustainable livelihoods analysis framework treats farmers as those who live or earn a living in vulnerable contexts, using whatever capital they have to maintain their livelihoods as much as possible. This section, again starting from a sustainable livelihoods analysis framework, constructs a model of the determinants affecting farmers’ entrepreneurial income, including social capital, human capital, financial capital, political capital, and capabilities. Based on the fact that rural laborers mostly start their businesses in the form of small and microenterprises, with small production and operation scales and financial statistics that are not standardized, this chapter combines the contents of the CFPS 2010–2018 questionnaire design to select entrepreneurs among farmers and uses the last year’s net income of self-employed (private) operators as an indicator of entrepreneurial income. The key explanatory and control variables are presented in Table 1.

**Table 1:** Analysis of variables.

Variables	Specific variables	Variable symbols	Description
Dependent variable	Entrepreneurial income	Income	Last year, the net income of individual (private) operators, logarithmic treatment
Core explanatory variables	Endowed social capital (clan networks)	Clan	Whether there is a genealogy or clan shrine, no is 0, yes is 1
Control variables	Leapfrog social capital	Gift	All gifts given out last year
	Human capital	Age	Age
		Gender	Gender, female is 0, male is 1
		Education	Years of education
		Health	Health level, unhealthy is 0, healthy is1
	Financial capital	Financial	0–1
	Political capital	Political	1 for communist party members and participation in other associations, 0 for none
	Personal capabilities	Capability	Investigator’s comprehension, appearance, interpersonal skills, and language skills scores (values 1–7)

Compiled by the author.

Since the variable of social capital itself is not easy to measure, most scholars have conducted relevant studies on the variable of social networks (Dong et al., 2018). In this paper, we consider that social capital is divided into endowed social capital and leapfrog social capital. This section draws on the research of Wang and Zhou (2013) and Wang and He (2014) and assumes that due to the closed and open characteristics of the social network in which the individual acts, their social capital can be divided into integrated and leapfrog. Following the research theme of this paper, this paper divides farmers’ social capital into endowed-type and leapfrog-type social capital. The former is an innate social network and does not require specialized management, and this chapter refers specifically to clan networks. In such networks, clan members can fully exchange information with each other because of the relatively closed and emotional nature of the member relationships. As Peng (2009) argues, the revived clan is no longer a legal organizational entity but a collective action group, i.e., endowed with natural social capital because the clan network is bounded by blood and geographical ties, and within a clan village community, the clan does not exclude families within the village community. Similarly, each family cannot expel its family members. This relatively fixed-member structure is conducive to collective action and normative control. This kind of social capital belongs to what Granovetter called strong relational social capital.

Leapfrog social capital, on the other hand, is in an open network that requires individuals to expand outward to operate to reach a strong and reliable relationship. In the former network, relatives and friends do not necessarily need to maintain relational networks through gifts (Zhao and Lu, 2010) while in the latter network, farmers’ entrepreneurship requires gifts to maintain friends or relatives related to entrepreneurship, and such networks may be weakly relational and weakly connected. Based on the above analysis, this section divides social capital into endowed social capital and leapfrog social capital that need to be operated, which play different roles in the marketization process and have different impacts on farmers’ entrepreneurial income. At the same time, other livelihood capital variables (such as human capital, material capital, and financial capital) are controlled to analyze the impact of key variables on farmers’ entrepreneurial income.

Endowed social capital (clan networks): refer to Guo and Yao (2013), using “whether the village has a clan shrine or genealogy” to indicate the cohesiveness of the clan network as a proxy variable for the clan networks.

Leapfrog social capital: Social capital is usually measured by behavioral indicators such as “the number of people who can help when looking for a job” (Chen et al., 2009) and “the exchange of gifts between friends and relatives” (Ma and Yang, 2011; Wang and He, 2014). In the former clan networks, relatives and friends do not necessarily need to maintain relational networks through gifts (Zhao and Lu, 2010), while in the latter network, farmers need to maintain gifts for entrepreneurship mainly for friends related to entrepreneurship. Therefore, this paper uses “gift exchange between friends and relatives” as a proxy indicator to measure leapfrog social capital. The measure can reflect the content of social capital and the degree of mutual assistance among farmers.

Human capital includes years of education and health level (self-assessed whether healthy or unhealthy). Political capital refers to the legal spillover effects of political resources (Feng, 2022) that peasant entrepreneurs can use to increase their entrepreneurial income and obtain stronger financing and sales competitiveness. In the Chinese context, individuals are considered to have political capital if they join the Communist Party of China or other political parties; otherwise, they are considered not to have political capital.

Financial capital is characterized by credit availability (Li et al., 2020), and the questionnaire is as follows. When borrowing large amounts of money (e.g., for buying a house, business turnover, etc.); if it is rejected, who has rejected it? (1, Relatives; 2, Friends; 3, Banks; 4, Nonbank formal financial institutions; 5, Private lending institutions and individuals). Since this question was not available in the 2010 and 2012 questionnaires, the data for these 2 years will be populated according to the trend. Then, the average of the results is processed, and the result is between 0 and 1. The closer the value is to 1, the stronger the credit constraint of the sample. Ability variables are limited by the availability of questionnaire data, and this chapter focuses on the total mean of respondents’ comprehension, appearance, interpersonal level, and verbal ability as a proxy variable for entrepreneurial ability. The relevant questions covered by the 2018 research questionnaire were the respondent’s appearance and the respondent’s intelligence level, so the 2018 competencies were only based on these two.

Some of the missing values were added in the data cleaning. Table 2 shows the results of descriptive statistics for the variables covered.

**Table 2:** Summary statistics.

Variables	Obs.	Mean	Std.	Min.	Max.
Ln_income	4,485	8.873	1.511	0.693	13.42
Clan	4,485	0.334	0.472	0	1
Gift	4,485	4,078	6,719	0	150,000
Age	4,485	48.23	9.840	19	64
Gender	4,485	0.491	0.500	0	1
Education	4,485	3.109	2.681	0	16
Health	4,485	0.822	0.383	0	1
Political	4,485	0.0630	0.243	0	1
Financial	4,485	0.0670	0.136	0	1
Capability	4,485	5.060	1.213	1	7
Market	4,485	2.974	0.0950	2.830	3.440

Author’s compilation based on CFPS data.

### Methodology

The main empirical is to obtain the coefficients of the entrepreneurial income determination equation by regression, focusing first on the income gap of farmers’ entrepreneurship, referring to Mincer (1974) human capital return equation and improving it. The entrepreneurial income determination Equation 1 is as follows:


(1) $$\ln\left(Y\right)=\alpha +\sum \beta iXi+\lambda \delta (\gamma^{\prime }w)/\phi (\gamma^{\prime }w)+\varepsilon$$

In the above equation, Y represents the income of farmers’ entrepreneurship and is the corresponding explanatory variable, α is the constant term and is the corresponding explanatory variable, including endowed social capital, leapfrog social capital (Munshi and Rosenzweig, 2016), age, gender (Chantreuil and Lebon, 2015), human capital (Gao and Yao, 2006), political capital (Feng, 2022), financial capital (Li et al., 2020), and personal ability (Yu et al., 2020). (γʹ w)/ф(γʹ w) is the correction term, and ε is the portion of income that cannot be explained by the correction term.

Since the equation that determines farmers’ entrepreneurial income is a semilogarithmic model, using a direct logarithmic decomposition of total farmers’ entrepreneurial income would distort the distribution of the attribute variables (Zhao and Lu, 2010). Therefore, the equation needs to be transformed before decomposition, see the Equation 2:


(2) $$Y=\exp\left(\alpha +\sum \beta iXi+\lambda \delta (\gamma^{\prime }w)/\phi (\gamma^{\prime }w)+\varepsilon \right)$$

### Semilogarithmic model

A semilog model is a model in which one of the dependent variables and the explanatory variable are in logarithmic form and the others are linear. The dependent variable in logarithmic form is a log-linear model (log-lin model), and the explanatory variable in logarithmic form is called a linear-log model (lin-log model). In this paper, the former form is used, and the model is:


(3) $$\ln Y_{t}=\beta_{0}+\beta_{1}X_{t}+\mu_{t}.$$

The percentage change in the dependent variable Y caused by a one unit change in the explanatory variable X. Multiplying this relative change by 100 gives the percentage change in Y, which is the growth rate of Y. Because of this meaning of the slope coefficient in the log-linear model, it is also called the growth model.

The semilogarithmic model is chosen for the income determination equation because comparisons of various forms of income equations have been made in the previous literature, and the results show that the semilogarithmic model either outperforms or does not differ significantly from the other models in terms of goodness of fit (Wan, 2004). Considering the treatment of the constant term in the decomposition process, the contribution of the constant term to the income gap becomes more difficult to handle if a fully linear model is used. Theoretically, whether the constant term has a contribution to the income gap is controversial, while if a semilogarithmic model is used, the constant term will be transformed into a constant product term in the equation to be decomposed, which has no effect on the contribution to the income gap; therefore, using a semilogarithmic model can also avoid the controversy of whether the constant term has a contribution to the income gap and is a better choice for the model.

### Quantile regression method

In general, traditional regression analysis studies the relationship between the independent variable and the conditional expectation of the dependent variable, and the corresponding regression model obtained can estimate the conditional expectation of the dependent variable from that of the independent variable. Quantile regression studies the relationship between the independent variable and the conditional quantile of the dependent variable, and the corresponding regression model obtained can estimate the conditional quantile of the dependent variable from that of the independent variable. Compared with the traditional regression analysis that can only obtain the central trend of the dependent variable, quantile regression can further infer the conditional probability distribution of the dependent variable. According to the different characteristics of the regression parameters, quantile regression models can be classified into three categories: parametric regression models, non-parametric regression models, and semiparametric regression models.

The quantile regression method is a fitted regression of the dependent variable on the independent variable through the conditional distribution of the dependent variable, which is a structural extension analysis and global analysis of the OLS mean regression, and the results show the changes and effects of the independent variable on the local distribution characteristics of the dependent variable. Then, because of the better analysis of the tail characteristics of the distribution of the dependent variable, richer information generated with the changes in the distribution of the dependent variable can be obtained, which is conducive to inductive summary of the change law, so the quantile regression method is widely used in income-related studies. The quantile regression method helps to understand the degree of influence of clan networks on farmers’ entrepreneurial income at different quartiles.

### Propensity score matching method

There are several main sources of endogeneity: reverse causality (also known as simultaneity bias), omitted variables, measurement error, sample selection bias, and model setting bias. When dealing with endogeneity, econometrics generally consider the instrumental variables approach, quasi-natural experiments, or propensity score matching (PSM).

The PSM can not only solve the self-selection, but also can eliminate the bias due to the improper setting of the model functional form (FMM) (Shipman et al., 2016; Lian et al., 2020), mainly is by reducing the reliance on the functional form specification. The PSM is used to perform robustness tests by pairing the samples and then estimating the measures. This method was first proposed and applied by Heckman et al. (1998). The core idea is to find a suitable counterfactual control group (i.e., entrepreneurial farmers who did not have a clan network) for the treatment group (i.e., entrepreneurial farmers who had a clan network).

The specific steps are as follows: based on those observable individual and household characteristics, find the entrepreneurial farmers who do not have clan networks with similar characteristics to those who have clan network entrepreneurial farmers as counterfactuals, and then calculate the entrepreneurial income gap between the two groups according to different methods to obtain the average treatment effect of clan networks on farmers’ entrepreneurial income (Average Treatment Effect on Treated). See Equation 4 for details:


(4) $$\begin{array}{l} \tau_{ATT}=\\ E_{p\left(X\right)D=1}\left[E\left(Income_{1}-Income_{0}\right)|D=1\mathrm{|,}p\left(X\right)\right]=\\ E_{p\left(X\right)|D=1}\left[\begin{array}{l} E\left(Income_{1}|D=1,p\left(X\right)\right)-\\ E\left(Income_{0}|D=1,p\left(X\right)\right) \end{array}\right]. \end{array}$$

The propensity value matching method requires that after satisfying certain preconditions, the $E\left(Income_{1}|D=0,p\left(X\right)\right)$ can be used instead of$E\left(Income_{0}|D=1,p\left(X\right)\right)$. The equation for using PSM estimators is given in Equation 5:


(5) $$\tau_{ATT}^{PSM}=E_{p\left(X\right)|D=1}\left[\begin{array}{l} E\left(Income_{1}|D=1\mathrm{|,}p\left(X\right)\right)-\\ E\left(Income_{0}|D=0,p\left(X\right)\right) \end{array}\right].$$

PSM is subject to two prerequisites: first, the conditional independence assumption, i.e., after controlling for the presence or absence of a clan network state (D), i.e., Income⊥D|p(X); and second, the density function passes the support assumption (Common Support, satisfying 0<p(X)=$P_{r}$(D = 1|X) < 1)). The conditional independence assumption requires that the estimation needs to control for as many factors as possible that have an impact on farmers’ entrepreneurial income based on previous literature. At the same time, sample matching is also required after calculating the propensity score, and a balance test is needed.

## Empirical results and discussion

### Analysis of basic model regression results

There may be multi-collinearity among the various types of capital in the model, so before the empirical evidence, we first check the value of the variance inflation factor (VIF). Generally, if VIF exceeds 10, the regression model has serious multi-collinearity, which means the model is poorly constructed. If all of them are less than 10 (strictly speaking, it should be 5), then the model has no multi-collinearity, and the model is well constructed. The mean value of VIF of the variables is 1.1, which indicates that the model does not have the multi-collinearity problem.

Based on the above model, the regression results are presented in Table 3. Column 1, as the estimation result of the baseline equation, shows that the coefficient estimate of clan network is positive and statistically significant at the 1% level, i.e., if farmers have a clan network, entrepreneurial income will significantly increase. That is, the clan network, an endowed social capital, significantly and positively affects farmers’ entrepreneurial income.

**Table 3:** The effect of clan on farmers’ entrepreneurial income.

Variables	(1)	(2)	(3)	(4)	(5)
Clan	0.137*** (0.005)	0.116** (0.018)	4.585*** (0.003)	0.140*** (0.004)	3.233** (0.042)
Gift	0.000*** (0.000)	0.000*** (0.000)	0.000*** (0.000)	0.000*** (0.000)	0.000*** (0.000)
Age	−0.022*** (0.000)	−0.023*** (0.000)	−0.023*** (0.000)	−0.025*** (0.000)	−0.028*** (0.000)
Gender	0.308*** (0.000)	0.312*** (0.000)	0.313*** (0.000)	0.304*** (0.000)	0.303*** (0.000)
Education	−0.006 (0.427)	−0.014* (0.087)	−0.015* (0.077)	0.046*** (0.000)	0.039*** (0.001)
Health	0.148*** (0.010)	0.132** (0.021)	0.129** (0.024)	0.155*** (0.007)	0.131** (0.021)
Political	−0.068 (0.447)	−0.046 (0.605)	−0.046 (0.607)	−0.071 (0.431)	−0.028 (0.757)
Financial	0.557*** (0.001)	0.633*** (0.000)	0.640*** (0.000)	0.539*** (0.002)	0.653*** (0.000)
Capability	0.026 (0.132)	0.029* (0.094)	0.026 (0.126)	0.018 (0.290)	0.019 (0.275)
Market		1.212*** (0.000)	1.654*** (0.000)		
Ins			−1.500*** (0.004)		−1.055** (0.048)
Year dummies	No	No	No	Yes	Yes
Province dummies	No	No	No	No	Yes
*N*	4,485	4,485	4,485	4,485	4,485

*, **, and *** represent significance at the 10, 5, and 1% levels, respectively.

The leapfrog social capital of farmers also directly affects the entrepreneurial income of farmers as follows: the clan network, as farmers’ endowment capital, is inherent without any special management afterward. This kind of relationship originates from social mobility and needs to be operated by farmers and members of different social groups, which is represented by human gifts, mostly to friends and colleagues, and extended to form the leapfrog social capital variable. When the farmer entrepreneur gives out more human gifts, it brings various benefits, such as trust and mutual assistance, forming a more effective social interaction mechanism. The more social capital they accumulate, the better their ability to identify entrepreneurial opportunities, and the more information they obtain through their networks, which leads to an increase in entrepreneurial income. This conclusion is similar to Wang and He (2014), who argues that leapfrog social capital positively supports migrant workers’ income.

The social network relations of farmers are characterized by both endowment type and leapfrog type, and the two types of social capital play different roles in farmers’ entrepreneurship. Endowed social capital may run through the whole process of farmers’ entrepreneurship, playing the role of serving as an information channel, reducing transaction costs and thus enhancing entrepreneurial income, but the process of entrepreneurship requires the development of leapfrog-type social capital, which has a positive effect on farmers’ further expansion of entrepreneurship. These two types of social capital together support farmers’ entrepreneurial income enhancement.

Columns 2 and 3 in Table 3 add the market index variable and the cross-multiplier variable between the informal and market systems, respectively. The regression results in Column 2 indicate that after adding the market index variable, clan network social capital is still significantly positive at the 5% level, with a decrease in the coefficient, and the market index variable significantly and positively affects farmers’ entrepreneurial income at the 1% level, indicating that a better entrepreneurial market environment can promote higher entrepreneurial income. After adding the market variable, the market variable is significantly positive, but the significant coefficient of the clan network variable becomes significantly smaller, indicating that market power gradually increases at this stage and that the formal institution has more influence on farmers’ entrepreneurial income than the informal institution of the clan network. This indicates that the advancement of the formal system produced a vacuum contraction effect of the informal system and that the influence of the clan network was skewed to a lesser extent. However, after adding the cross product term in Column 3, the market variable is still significantly positive at the 1% level, and the clan network also plays an influential role. Considering that the wave of mass entrepreneurship was proposed and launched in 2014, the time factor of policy support may affect entrepreneurial income, so the time factor is controlled in Column 4.

The results show that the clan network still positively affects farmers’ entrepreneurial income. Since the observable phenomenon of territorial entrepreneurship is obvious, the influencing factors of entrepreneurial income should also consider regional characteristics. After taking into account time and region in Column 5, the clan network is significantly positive.

The results show that the clan network still positively affects farmers’ entrepreneurial income. Since the observable phenomenon of territorial entrepreneurship is obvious, the influencing factors of entrepreneurial income should also consider regional characteristics. After taking into account time and region in Column 5, the clan network is significantly positive.

The effects of other explanatory variables on farmers’ entrepreneurial income are briefly explained here. First, the age variable significantly and negatively affects entrepreneurial income, which is more consistent with many study findings, suggesting that older individuals no longer have an entrepreneurial advantage. After taking into account the time trend, increasing educational attainment significantly increases entrepreneurial income. Health significantly and positively affects farmers’ entrepreneurial income levels, and the healthier the farmer is, the more successful he or she will be in entrepreneurship, which is also consistent with everyday perceptions.

### Robustness checks

**Figure 1:**
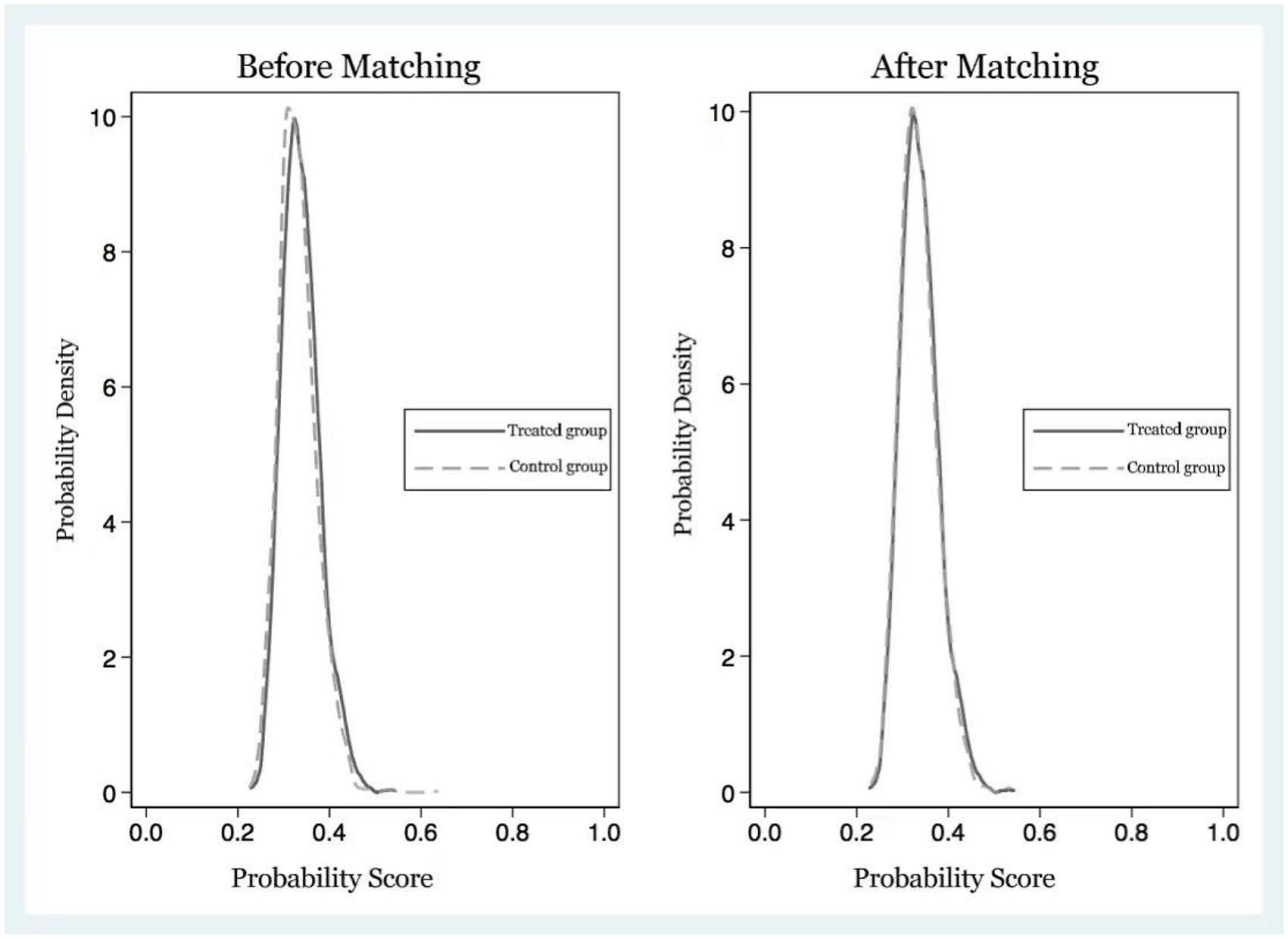
P-score fit before and after overall sample matching.

As analyzed in the previous section, the PSM method not only solves the self-selection problem, it also alleviates the FFM problem by reducing the dependence on the functional form setting, which plays an important role in reducing the correlation between treatment variables and observable variables to solve the endogeneity problem. Therefore, in the robustness analysis, we uses the PSM method to analyze the effect of the presence of farmers’ clan networks on farmers’ entrepreneurial income. According to the study, the analysis using the PSM method should satisfy the parallelism assumption and the common support assumption. Figure 1 presents the kernel density distribution of propensity matching score values (P-Score) for the treatment and control groups for the overall sample of panel data before and after matching. Before matching, the center of gravity of the propensity matching score distribution for the control group is significantly higher than that of the treatment group and left-skewed. After matching, the center of gravity of the propensity matching score distribution of the control group shifted to the right and increased the overlap with the center of gravity of the propensity matching score distribution of the treatment group. The convergence of the propensity matching score distributions of the treatment and control groups were similar, and the difference between the propensity matching scores of the two groups was significantly corrected to meet the common support hypothesis.

Then, the sample matching variables and the balance test were conducted (see Table 4 for details). The equilibrium test requires that the variables do not differ significantly between the treatment and control groups after matching is conducted, and the matching effect is determined mainly by the absolute value of the post-match *t* value and the post-match standard deviation. A small absolute value of the t-statistic indicates that there is no systematic difference between the two groups of characteristic variables after matching. According to Dehejia and Wahba (2002), the absolute value of the standard deviation after matching should be less than 5%.

**Table 4:** Sample matching variables and balance test.

Matching variables		Mean value		Standard deviation (%)	Error abatement (%)	*t*-test	
		Treated group	Control group			*t*	*p* > *t*
Gift	Before matching	4308.4	3962.9	5.2		1.62	0.104
	After matching	4308.4	4095.3	3.2	38.3	0.90	0.370
Age	Before matching	47.699	48.499	−8.2		−2.57	0.010
	After matching	47.699	47.549	1.5	81.3	0.41	0.680
Gender	Before matching	0.49666	0.48879	1.6		0.50	0.619
	After matching	0.49666	0.51638	−3.9	−150.7	−0.77	0.443
Education	Before matching	3.3496	2.9885	13.3		4.26	0.000
	After matching	3.3496	3.3469	0.1	99.3	0.03	0.980
Health	Before matching	0.83021	0.81766	3.3		1.04	0.301
	After matching	0.83021	0.83055	−0.1	97.3	−0.02	0.981
Political	Before matching	0.06885	0.06022	3.5		1.12	0.263
	After matching	0.06885	0.06484	1.6	53.5	0.44	0.661
Financial	Before matching	0.06537	0.06711	−1.3		−0.40	0.686
	After matching	0.06537	0.06571	−0.2	80.8	−0.07	0.945
Capability	Before matching	5.1656	5.0064	13.2		4.15	0.000
	After matching	5.1656	5.1833	−1.5	88.9	−0.42	0.678

Table 4 shows that after matching, the standardized deviations of all variables in the control and control groups were less than 5%, and the results of the t test did not reject the original hypothesis of no systematic differences between the control and control groups, i.e., they passed the balance test of PSM.

After satisfying the above two prerequisites, the entrepreneurial farmers with clan networks were used as the treatment group and the other farmers as the control group according to the definition above. The PSM method using the k-nearest neighbor matching method, nearest neighbor matching method within caliper and kernel matching method was further employed to match the experimental and control groups. It was found that the estimation results of the three methods were closer in the case of large samples. The mean treatment effects were calculated based on the matched samples, and the results can be seen in Table 5.

**Table 5:** Estimated impact of clan networks on farmers’ entrepreneurial income.

Matching method	Treated group	Control group	Average treatment effect	Standard deviation
k-nearest neighbor matching	8.981	8.873	0.108	0.058*
Caliper matching	8.981	8.866	0.115	0.048**
Kernel matching	8.981	8.842	0.139	0.048***
Mean value	–	–	0.121	–

*, **, and *** represent significance at the 10, 5, and 1% levels, respectively.

The mean treatment effects for the experimental, control and overall samples were significantly positive at the 10% level (k-nearest neighbor matching method), 5% level (caliper matching method) and 1% level (kernel matching method), i.e., after controlling for initial differences in conditions using PSM, farmers’ ownership of clan networks significantly increased entrepreneurial income, indicating that the results of the analysis are more robust.

### Gender classification analysis

Since previous studies have considered the effect of gender differences on entrepreneurial income (Chen et al., 2015; Li and Yang, 2020), a gender disaggregated analysis is necessary. The results of the subgroup estimation for males and females can be found in Table 6, which shows that the clan network has a significant effect on entrepreneurial income for both males and females, but the effect is greater for females. As the main performer of rituals in the clan network, men’s clan network is a strong relationship, which reflects the higher degree of social connection and interaction among clan network members, and they are more likely to use this endowed social capital to make connections in entrepreneurship.

**Table 6:** Estimated results of the income determination equation for farmers’ entrepreneurship (by gender).

Variables	Male	Female
Clan	0.122* (0.076)	0.148** (0.035)
Gift	0.000*** (0.000)	0.000*** (0.000)
Age	−0.021*** (0.000)	−0.023*** (0.000)
Education	−0.007 (0.488)	−0.004 (0.802)
Health	0.118 (0.186)	0.166** (0.027)
Political	−0.150 (0.125)	0.282 (0.174)
Financial	0.455* (0.065)	0.644*** (0.007)
Capability	0.034 (0.147)	0.017 (0.503)
*N*	2,204	2,281

*, **, and *** represent significance at the 10, 5, and 1% levels, respectively.

With the acceleration of China’s economic development and marketization, the influence of clan networks is no longer limited to male members but is also manifested in the contemporary rise of women’s status, such as women’s access to genealogy, and the important role of women in clan networks, which is also more common in actual research. Moreover, according to Lin and Zhang (2005), this may be related to the fact that rural women have less social capital accumulation, women’s entrepreneurship is mostly small-scale, and they will certainly seize the clan network as an important source of innovative information and resources when starting a business. This is close to the conclusion that the institutional environment enhances the role of social capital, as studied by Li et al. (2017).

### Analysis of quantile regression results

The estimated results of the above study illustrate that the clan network, a form of social capital, has a significant role in farmers’ entrepreneurial income that cannot be ignored. However, is the effect of farmers’ entrepreneurial income consistent across quantile levels? To answer this question, this section further elaborates the effect of clan networks on farmers’ entrepreneurial income using quantile regression of the entrepreneurial income decision equation. Through the quantile regression method, four quartiles are selected in this chapter: 25% (low income), 50% (middle income), 75% (middle and high income) and 90% (high income). Table 7 present the quantile regression estimation results.

**Table 7:** Quantile regression estimation results for farmers’ entrepreneurial income.

Variables	(1) 25%	(2) 50%	(3) 75%	(4) 90%
Clan	0.129* (0.067)	0.203*** (0.000)	0.104* (0.069)	0.044 (0.419)
Gift	0.000*** (0.000)	0.000*** (0.000)	0.000*** (0.000)	0.000*** (0.000)
Age	−0.024*** (0.000)	−0.028*** (0.000)	−0.024*** (0.000)	−0.020*** (0.000)
Gender	0.322*** (0.000)	0.336*** (0.000)	0.243*** (0.000)	0.039 (0.464)
Education	0.057*** (0.000)	0.035*** (0.002)	0.025** (0.033)	0.037*** (0.001)
Health	0.044 (0.625)	0.157** (0.025)	0.207*** (0.005)	0.227*** (0.001)
Political	−0.147 (0.308)	−0.130 (0.250)	−0.176 (0.134)	−0.121 (0.282)
Financial	0.525** (0.032)	0.260 (0.173)	0.200 (0.314)	0.560*** (0.003)
Capability	0.020 (0.468)	0.049** (0.025)	0.063*** (0.005)	0.071*** (0.001)
*N*	4,485	4,485	4,485	4,485

*, **, and *** represent significance at the 10, 5, and 1% levels, respectively.

The quantile regression results show that the effect of clan networks on the increase in farmers’ entrepreneurial income varies across income quantiles. The effect of endowed social capital is most significant for the middle-and the middle-and higher-income end groups with larger coefficients. This is consistent with the findings of Grootaert (2001) through the use of quantile regressions, where the returns to social capital decrease as income increases across groups.

The table shows that the clan network, an endowed form of social capital, can be considered the social capital of the poor to some extent. In general, the social capital of the poor emphasizes that social capital is more favorable to the poor or people in poor areas, and the more social capital owned by the poor, or the more and greater returns to their ownership of social capital, is beneficial in alleviating the income gap between the rich and the poor. However, it is more important to see that endowed social capital has the greatest impact on middle-income groups. Leapfrog social capital has a significant positive effect on all groups.

## Conclusion

By analyzing the determinants and connotations of farmers’ entrepreneurial income, the influence of formal and informal institutions on the trajectory of economic development is revealed. A model of entrepreneurial income determination based on different capital types is constructed, with clan social capital as a farmer entrepreneurship influencing factor. It also classifies farmers’ social capital as established endowed social capital such as clan networks and leapfrogging social capital that supports business. In the marketization process, the two types of social capital play different roles and have different effects on farmers’ entrepreneurial income.

Specifically, the main findings are as follows. A semilogit regression model is applied as the main estimation strategy using CFPS 2010–2018 panel data, while a PSM method is used for robustness testing. The results show that the clan network, as an informal institution, shows an inverted U-shaped curve relationship with marketization and economic development level, and when the market institution gradually strengthens, the informal institution clan network shows a gradual fading trend in economic activities. After adding the market variable, the influence of the informal system of the clan network is weakened. The market system, on the other hand, significantly and positively affects farmers’ entrepreneurial income. After controlling for temporal and regional variables, the clan network still positively affects farmers’ entrepreneurial income at the 5% level.

First, this paper provides an empirical basis for the micro formation of the income gap in farmers’ entrepreneurship, showing that different types of social capital are important factors affecting entrepreneurial income. What needs to be recognized is that despite the revival of rural clans, with the development of urban–rural integration and the accelerated development of information digitization, farmers’ social network relationships carry both endowment-type and leapfrog-type characteristics, and the two types of social capital play different roles in farmers’ entrepreneurship. Endowed social capital may be the one that runs through the whole process of farmers’ entrepreneurship, playing the role of information channels, reducing transaction costs and enhancing entrepreneurial income. However, in the process it is necessary to develop leapfrog social capital, which has a positive effect on farmers’ further expansion of entrepreneurial scale. These two types of social capital together support farmers’ entrepreneurial income enhancement.

Second, the clan network, an endowed social capital, has a significant effect on entrepreneurial income for both men and women, but the effect is greater for women. The clan network has the greatest impact on middle-income farmers. This suggests to some extent that endowed social capital is more social capital for middle-income people and has a significant positive effect on the general income group.

## Policy implications and theoretical implication

### Policy implications

Analyzing the influencing factors affecting farmers’ entrepreneurial income is of practical significance for implementing rural revitalization strategies, improving farmers’ socioeconomic status, and promoting rural economic growth. The first policy implication is to focus on the role of clan networks on farmers’ entrepreneurial income, at the same time, to encourage farmers to develop leapfrog social capital through various channels so that both types of social capital can jointly support the increase in entrepreneurial income.

The second implication is to recognize that although the informal system of the clan network still plays a positive role, it is still inherently deficient in terms of incentives and constraints and access to resources compared with the formal system, while the formal market system represented by the market index has a significant positive effect on farmers’ entrepreneurial income. Therefore, a fair and open market system needs to be further developed and improved.

Third, the social capital function of clan networks still has resource and capacity attributes in contemporary times and has a positive effect on relieving the farmers’ entrepreneurship difficulties. The traditional clan culture has a strong villagers’ spirit, focusing on educating the village, dealing with the people and benefiting the people. As influential people with certain knowledge, skills, wealth, social status and cultural levels within the village, new country squire are an important social capital for ordinary farmers. In view of the fact that ordinary farmers usually face the problems of insufficient capital, lack of technical knowledge and high market risks in starting their own businesses, farmers can be guided to cooperate with new country squire in starting their own businesses. Compared with villagers, new country squire hold certain social resources and social capital, so using new country squire to lead the entrepreneurial tide can bring back capital, technology and advanced development and management concepts to the countryside. They can bring back capital, technology and advanced development and management concepts to the villages and inject new vitality into rural revitalization.

The local governments should actively make use of the spirit of new country worthy inherited from clans, make various types of new country worthy who have gone out and established their careers in cities willing to come back to hometown and promote the realization of ecological product value and common prosperity. Therefore, the local government should establish a database of new country squire, play the leading role of new country squire on farmers’ entrepreneurship, and should master different types of cooperation projects, choose different support models for different types of cooperation, for new country squire can work closely with farmers entrepreneurship projects to focus on support, to give policy, financial subsidies on the support.

### Theoretical implication

First, this paper provides an empirical basis for the micro formation of the income gap in farmers’ entrepreneurship, showing that different types of social capital are important factors affecting entrepreneurial income. What needs to be recognized is that despite the revival of rural clans, with the development of urban–rural integration and the accelerated development of information digitization, farmers’ social network relationships carry both endowment-type and leapfrog-type characteristics, and the two types of social capital play different roles in farmers’ entrepreneurship. Endowed social capital may be the one that runs through the whole process of farmers’ entrepreneurship, playing the role of information channels, reducing transaction costs and enhancing entrepreneurial income. However, in the process it is necessary to develop leapfrog social capital, which has a positive effect on farmers’ further expansion of entrepreneurial scale. These two types of social capital together support farmers’ entrepreneurial income enhancement.

Second, it is necessary to examine the impact of formal and informal systems on farmers’ entrepreneurial income. Although the clan network as an informal system to some extent compensates for the shortcomings of the formal system and promotes both farmers’ entrepreneurial entry decisions and entrepreneurial income, the informal system is still a suboptimal choice. The study findings also suggest that the formal market system has a more pronounced effect on promoting farmers’ entrepreneurial income. This is because the informal system is congenitally deficient in terms of incentives and constraints relative to the formal system, and there is the phenomenon that only those entrepreneurial enterprises that can use family ties to access core resources can use economic resources within the system at low cost, while grassroots entrepreneurship has more difficulty accessing resources, and unfair competition inhibits private entrepreneurship. In practice, we cannot ignore the negative impacts and effects of clan networks, such as possible market monopoly, or the failure of entrepreneurship within the entire clan group due to poor decision-making by the first action group.

The formal market system has a more powerful role in promoting farmers’ entrepreneurship. Therefore, a high-level market economy system should be built within the comprehensive deepening reform, focusing on stimulating the vitality of market players and improving the fair competition system, property rights and intellectual property protection systems to further protect the development of private enterprises and the private economy, thus ensuring the vitality and high-quality development of the economy.

## Data availability statement

The raw data supporting the conclusions of this article will be made available by the authors, without undue reservation.

## Ethics statement

The informed consent of the participants was implied through survey completion. An ethics approval was not required as per applicable institutional and national guidelines and regulations.

## Author contributions

XJ and QW: data curation. XJ, XM, and BJ: methodology. XJ and LW: writing—original draft. XJ and XM: writing—review and editing. All authors contributed to the article and approved the submitted version.

## Funding

This study was supported by the Fujian Innovation Strategy Research Project (2022R0106); Open Foundation of Xi Jinping Thought on Socialism With Chinese Characteristics for the New Era, Minjiang University (JYLS2021003); Introduction of Talents and Social Science Project of Minjiang University (MJY21045); Fujian Province Xi Jinping Thought on Socialism With Chinese Characteristics for the New Era Research Center Project (FJ2021XZB028); and Fujian Province Young and Middle-Aged Teachers Education Research Project (Social Science; JAS21263).

## Conflict of interest

The authors declare that the research was conducted in the absence of any commercial or financial relationships that could be construed as a potential conflict of interest.

## Publisher’s note

All claims expressed in this article are solely those of the authors and do not necessarily represent those of their affiliated organizations, or those of the publisher, the editors and the reviewers. Any product that may be evaluated in this article, or claim that may be made by its manufacturer, is not guaranteed or endorsed by the publisher.
